# Editorial: New Horizons in Health-Promoting: From Methods to Implementation Science

**DOI:** 10.3389/fphar.2021.830957

**Published:** 2022-01-14

**Authors:** Silvio Barberato-Filho, Cristiane de Cássia Bergamaschi, Brian Godman, Marcus Tolentino Silva, Fernando de Sá Del Fiol, André Oliveira Baldoni, Jorge Otávio Maia Barreto, Luciane Cruz Lopes

**Affiliations:** ^1^ Pharmaceutical Science Graduate Course, University of Sorocaba (Uniso), Sorocaba, Brazil; ^2^ Strathclyde Institute of Pharmacy and Biomedical Sciences, University of Strathclyde, Glasgow, United Kingdom; ^3^ Centre of Medical and Bio-allied Health Sciences Research, Ajman University, AlAin, United Arab Emirates; ^4^ School of Pharmacy, Sefako Makgatho Health Sciences University, Garankuwa, South Africa; ^5^ Oswaldo Cruz Foundation (Fiocruz), Rio de Janeiro, Brazil; ^6^ Universidade Federal de São João Del-Rei, São João Del Rei, Brazil

**Keywords:** health system, health care, health technology assessment, knowledge translation, implementation science, rational use of medicines, drug utilization research

New technologies with the potential to improve population health are being continually introduced through research and innovation. To improve care with available resources, it is necessary to promote the most cost-effective technologies, respecting organizational, social and ethical issues. This can be a concern in recent years with the growth in pharmaceutical expenditure being driven by new medicines for complex diseases, including cancer and orphan diseases, often though with limited health gain (Cohen, 2017; Luzzatto et al., 2018; Godman et al., 2021c; Moreira et al., 2021). This calls for greater evidence-based approaches to healthcare especially in countries with universal healthcare systems faced with rising costs and continued unmet need (Camargo et al., 2016; Fulone et al., 2016; Godman et al., 2021b).

Health Technology Assessment (HTA) has become an indispensable tool to inform health decisions and contribute to best practices, whether in the clinical or management scope. Furthermore, it has been widely used to improve health policies. This Research Topic seeks to debate in way the current challenges for the application of health technology innovation - specifically the use of rational and evidence-based approaches.

Publications contained in this Research Topic (n = 23 studies) summarized information regarding to health technologies (medicines, pharmaceutical services, health litigation, medical devices, diagnostics test, and other health interventions) focusing on reporting of therapeutic risk management (adverse drug reactions - ADRs, drug-drug interactions - DDIs, non-responding to treatment and other adverse outcomes (n = 4); available evidence of effectiveness and/or safety of medicines (n = 5); drug utilization research (n = 1); effectiveness of other interventions (services pharmaceutical and community-based interventions) (n = 2); methodological studies (n = 2); health litigation (n = 3); implementation science (n = 4); cost-effectiveness analysis (n = 1); and digital technologies (n = 1).

ADRs are a predominant public health problem especially among the elderly, hospitalized and those patients on multiple medicines. Aspects related to safety regarding the use of medications among children were also studied (Calderon-Ospina et al.; Lima et al.) alongside the adults/elderly (Ferreira et al.). Another study analyzed adverse neonatal outcomes in adolescent pregnancy (Honorato et al.).

One study collected information from the database of the Brazilian Surveillance Agency, known as Notivisa, where reports of suspected serious adverse drug reactions (SADR) mainly with IV dosage forms and off label drugs, were observed demonstrating the need for further drug research in this area including the use of better dosage forms among children (Calderon-Ospina et al.). Another study collected data from pediatric intensive care units (PICU) in a teaching hospital. The authors observed that most of children were exposed to at least one potential DDI during PICU stay and there was increased of length of stay in children with major or contraindicated DDIs (Lima et al.).

Another study analyzed the prevalence of ADRs in a population with Alzheimer’s disease. Data were collected through interviews with patients and caregivers who obtained their medication for Alzheimer’s at a public pharmacy in Brazil, complemented by the prescribing data system. Most patients had at least one ADR, mainly due to psychiatric disorders (Ferreira et al.).

The risks of adverse neonatal outcomes in younger pregnant adolescents properly followed through group prenatal care, delivered by specialized public services, were also analyzed. Data were obtained from medical records and interviews with a multidisciplinary team that treated these patients. The lower incidence of adverse outcomes was observed in younger pregnancy adolescents that followed the prenatal care model (Honorato et al.). We believe this provides guidance to others experiencing such concerns.

The effectiveness in the use of medicines was also addressed in studies of Vieira et al., Fulone et al., Pedroso et al., Zhang et al., and Nascimento et al.

A systematic review compared the efficacy of five antiangiogenic drugs for the treatment of diabetic macular edema. The findings demonstrated the effectiveness of these medicines but there were differences between them for the outcomes evaluated. This study concludes that more randomized controlled trials are needed to confirm these findings Zhang et al.

Switching between second-generation antipsychotics (SGA) is a common clinical practice in the treatment of schizophrenia and schizoaffective disorders due to differences in the tolerability and safety profiles of the different anti-psychotics (Leucht et al., 2013; Huhn et al., 2019; Fulone et al., 2021b), as well as the challenge of obtaining an ideal response (Fulone et al., 2016; Mayer et al., 2021). Data were obtained from a pharmaceutical assistance program, by using an administrative database. The groups most susceptible to SGA switching were older individuals, women, and those living in the northeast region of Brazil (Fulone et al.). Risperidone was the antipsychotic associated with the highest risk of switching. Previous research conducted in Brazil has also shown greater effectiveness of olanzapine versus risperidone in routine clinical care (Barbosa et al., 2021).

De-escalation guided by blood cultures for patients with a diagnosis of sepsis, severe sepsis or septic shock were also analyzed to verify if blood cultures will reduce mortality and antimicrobial drug resistance in adults admitted to intensive care units (ICU). Overall, the hospital mortality was lower in the group who received empirical broad-spectrum antibiotic therapy (EAT), although the antibiotic therapy guided by blood cultures (ATGBC) also reduced the mortality in these patients (Pedroso et al.). Appropriate empiric use of antibiotics is important to reduce morbidity, mortality and costs associated with antimicrobial resistance (Godman et al., 2021).

Almost 30% of new antiepileptic drugs have drug-resistant forms of the disease. A cohort study assessed the impact of a number of genetic factors and their possible association with treatment response to phenytoin. Little more than half of the evaluated subjects were non-responding to phenytoin and the decreased function alleles of CYP2C9 were predictive of vestibular-cerebellar ADR to phenytoin (Vieira et al.). This is important to help guide future treatment approaches.

A 16-years cohort constructed by deterministic and probabilistic matching, through three databases of the Brazilian public health system, assessed the impact on liver graft survival with two different calcineurin inhibitors (tacrolimus or cyclosporine). Younger patients had a longer graft survival time than older ones. The use of tacrolimus was also more prevalent and had a better survival rate. Older age and the greater time ratio without using an immunosuppressant were identified risk factors for liver graft loss. White-skinned patients and tacrolimus-based regimens are factors for improved graft survival (Nascimento et al.).

The use of proton pump inhibitors (PPI) in ambulatory care was analyzed based on aggregated utilization data from the National Health Insurance database. Despite the tolerability and clinical benefits, potential long-term side effects have been observed with PPIs. There is a concern that patients receive PPIs in high doses and for a long period of time resulting in health authorities seeking to encourage rapid transfer from a healing to a maintenance dose. Overall, pantoprazole was the PPI most frequently used by hospitalized patients (Matuz et al.).

The DIATHEM observational study (type 2 DIAbetes: optimizing THErapy by Medication review in community pharmacies) evaluated the impact of medication reviews from community pharmacies in elderly patients with type 2 diabetes under real-life conditions. The study shows that medication reviews performed by community pharmacists reduced the frequency and number of drug-related problems even though the pharmacies had to face obstacles such as lack of cooperation by the prescriber or lack of reimbursement (Schindler et al.). This is important with the envisaged greater role of pharmacists to help with healthcare (Lima et al., 2008; Vieira et al., 2010; Yamauti et al., 2017).

Sustainable long-term interventions, such as health worker training and provision of health education to the patients, contribute to health improvements in patients with hypertension and diabetes from rural communities (Lemos Macedo et al.). This is important for the future especially if community pharmacists are the principal healthcare provider, and often the first point of contact for patients across countries (Marković-Peković et al., 2017).

Rosa et al. showed that using POC-A1c devices in primary care settings is a cost-effective alternative for monitoring glycated hemoglobin A1c as a marker of blood glucose control in people living with type 2 diabetes (Rosa et al.). This is important given rising prevalence rates for diabetes across countries with an appreciable impact on morbidity, mortality and costs if not properly managed (Araujo et al., 2016; Barros et al., 2020; Lam et al., 2020; Chan et al., 2021; Chan et al., 2021).

Two methodological studies addressed a newly developed instrument (HM-PRO) to evaluate patients’ health-related quality of life (HRQoL) with hematological malignancies and their symptom experience in daily clinical practice as well as in research (Goswami et al.;Goswami et al.). The findings can be used to improve the care of these patients in the future. Others initiatives can be used to increase knowledge translation and strengthening capacity core teams of health professionals (Tavares et al., 2019; Fulone et al., 2021a; Motter et al., 2021).

In Brazil, the Federal Constitution states that the right to health is a social right to be guaranteed by policies that promote and ensure universal and equal access to programs and services for the promotion, protection, and restoration of the health of citizens Yamauti et al. When policies do not guarantee these rights, the patients seek to the courts, characterizing the phenomenon known as the judicialization of health which can appreciable increase the cost of care. Three articles published in this research topic addressed this key issue. Silva et al. investigated the occurrence of serious adverse events related to medicines obtained by patients through health litigation and found that many of these medicines resulted in deaths and serious adverse events in expanded access and compassionate use programs in Brazil. Yamauti et al. summarized 78 strategies implemented by public institutions in Brazil to approach the judicialization of health care. Machado; Santos; Lopes et al. (2010) described the planning and implementation of strategies to approach medicines litigation at municipality level (Silva et al.; Yamauti et al.; Machado et al.). These papers are important to give guidance to the authorities in Brazil as they seek to tackle the increasing costs of litigation (Lopes et al., 2010).

Three qualitative studies emphasized the importance of considering patient preference for the development of guidelines and to develop strategies for behavioral change communication. Petrocchi et al. conducted a study with patients from Belgium and Italy undergoing treatment for lung cancer and found that aspects related to efficacy and safety, as well as psychological impact, are common areas of concern for patients regardless of origin cultural or country. Weller et al. contributed to understanding which factors influence patients’ adherence to venous leg ulcer treatment recommendations in primary care. Doke et al. studied the basic perceptions, knowledge, and behavior of women before the implementation of preconception care to reduce maternal and child mortality in India (Doke et al.; Petrocchi et al.; Weller et al.). Such approaches are essential to improve the health of patients.

The implementation of Clinical Practice Guidelines (CPGs) was also studied by Vasconcelos et al.. The authors concluded that even among high-quality CPGs, the implementation strategies report can be improved, especially concerning monitoring of implementation outcomes and selection of strategies based on relevant implementation barriers (Vasconcelos et al.). Studies have shown that multiple activities involved in the implementation and monitoring of adherence to guidelines helps enhance their use and impact (Godman et al., 2021b). In addition, the CPGs must be easy to use and regularly updated (Lopes et al., 2014; Silveira et al., 2014; Camargo et al., 2016; Niaz et al., 2020).

Digital health encompasses several technologies, which have the ability to monitor the human body continuously and uninterruptedly, collecting physiological data, and enabling the improvement of clinical practices. These technologies enable the integration of pharmacotherapies with self-care, lifestyle interventions and patient empowerment, while concurrently advancing patient-centered care, integrative medicine and digital health ecosystems (Bulaj et al.). This will be an increasing area of research as the use of technologies grows to monitor medicine use and outcomes across sectors.

In this Research Topic, a particular emphasis, was given to the technological development of new health care/services approaches describing processes regarding the introduction and implementation of technologies into health systems; the knowledge translation; evidence-based policy and its utility as a guide for implementation of health-promoting technologies; big data analytics for health policy in decisions making; and real-world cases. We will continue to monitor the situation to provide future guidance.
